# Managing Opioids and Mitigating Risk: A Survey of Attitudes, Confidence and Practices of Oncology Health Care Professionals

**DOI:** 10.3390/curroncol28010086

**Published:** 2021-02-12

**Authors:** Alissa Tedesco, Jocelyn Brown, Breffni Hannon, Lauren Hutton, Jenny Lau

**Affiliations:** 1 Temmy Latner Centre for Palliative Care, Sinai Health System, Toronto, ON M5T 3L9, Canada; 2 Department of Supportive Care, Princess Margaret Cancer Centre, University Health Network, Toronto, ON M5G 2C1, Canada; Jocelyn.Brown@uhn.ca (J.B.); Breffni.Hannon@uhn.ca (B.H.); Jenny.Lau@uhn.ca (J.L.); 3 Pharmacy Services, Queen Elizabeth II Health Science Centre, Nova Scotia Health, Halifax, NS B3H 2Y9, Canada; Lauren.Hutton@nshealth.ca

**Keywords:** opioid safety, opioids, addiction, oncology

## Abstract

In response to Canada’s opioid crisis, national strategies and guidelines have been developed but primarily focus on opioid use for chronic noncancer pain. Despite the well-established utility of opioids in cancer care, and the growing emphasis on early palliative care, little attention has been paid to opioid risk in this population, where evidence increasingly shows a higher risk of opioid-related harms than was previously thought. The primary objective of this study was to assess oncology clinicians’ attitudes, confidence, and practices in managing opioids in outpatients with cancer. This was explored using pilot-tested, profession-specific surveys for physicians/nurse practitioners, nurses and pharmacists. Descriptive analyses were conducted in aggregate and separately based on discipline. Univariate and multiple linear regression analyses were performed to explore relationships between confidence and practices within and across disciplines. The survey was distributed to approximately 400 clinicians in January 2019. Sixty-five responses (27 physicians/nurse practitioners, 31 nurses, 7 pharmacists) were received. Participants endorsed low confidence, differing attitudes, and limited and varied practice in managing and mitigating opioid risks in the cancer population. This study provides valuable insights into knowledge gaps and clinical practices of oncology healthcare professionals in managing opioids and mitigating associated risks for patients with cancer.

## 1. Introduction

In Canada, prescription opioid use has increased steadily over the past two decades with marked increases in the number of opioid overdoses and opioid-related deaths [1]. National strategies and guidelines have been developed to address the opioid crisis but they primarily focus on opioid use in chronic noncancer pain populations [2].

In oncology and palliative care, there remains a pervasive belief that the risk of opioid use disorder (OUD) is low, despite a paucity of supporting research [3,4]. Increasingly, evidence suggests that patients with cancer might be at higher risk of nonmedical opioid use (NMOU) than was previously thought [5,6,7]. An integrative literature review by Carmichael et al. found that at least one in five patients with cancer may be at risk of OUD [5]. Despite this, there is scant literature on opioid risk evaluation and mitigation in the cancer population, and few guidelines on how to manage NMOU and substance use disorders in patients with cancer [6,7,8,9]. The limited literature outlines similar risk mitigation strategies as those recommended for the chronic noncancer pain population including screening, a multidisciplinary approach, utilization of adjuvant agents, structured dispensing, pill counts, urine drug tests, and treatment contracts [10,11,12,13]. Unfortunately, the evidence base for utilizing these approaches for patients with cancer is lacking [13].

Given the increasing incidence and survival rates for cancer [14], and the growing emphasis on early palliative care [15], more patients are exposed to opioids for longer periods of time. It is thus essential that both clinicians and patients are educated around the safe and effective use of opioids, in order to maximize quality of life while minimizing risk. This study aimed to describe oncology clinicians’ attitudes, confidence, and practices around managing opioids prescribed to patients with cancer in the outpatient setting.

## 2. Materials and Methods

For this cross-sectional study, we developed three profession-specific surveys to assess opioid management and safety practices of those who provide outpatient clinical care, including: (1) physicians/nurse practitioners (MD/NP), (2) nurses (including registered nurses (RN) and clinical nurse specialists (CNS)), and (3) pharmacists. These surveys were developed by an interdisciplinary research team of nurses, pharmacists and physicians working in palliative care, based on clinical experience and nonsystematic literature review. The surveys were also reviewed by addictions medicine and oncology clinicians. There were no major differences in survey content between the three profession-specific surveys, however some questions were reworded or reframed in order to be relevant to the scope of each profession’s practice. A copy of the survey adapted for pharmacists is available in Supplementary Materials.

Each survey was piloted with at least two individuals from each profession. No major revisions to the survey content were necessary based on the feedback from this initial pilot. The surveys addressed the following topics: demographics, attitudes towards opioid use in cancer care, confidence and practices in managing opioids in cancer care, personal experiences with adverse events in patients with cancer using opioids, and education and resources. The surveys employed 4 and 5-point Likert scales to rate the frequency of practice, or level of agreement, respectively. Surveys were administered in English. Ethics approval was obtained through University Health Network—Research Ethics Board (Coordinated Approval Process for Clinical Research, ID 18-6052) on December 14, 2018.

Final surveys were built using Qualtrics Survey Software™ (Qualtrics XM, Qualtrics LLC, Provo, UT, USA), a secure web-based survey tool. Invitations were distributed in January 2019 by department leads in the areas of palliative care, oncology, nursing, and pharmacy at the Princess Margaret Cancer Centre (PM) in Toronto, Canada, a tertiary cancer center. Reminder emails were sent 4 weeks after the initial invitation emails were sent. The survey remained open for approximately 2 months. Eligible participants were RN, CNS, pharmacists, MD and NP fluent in English, and employed at PM to provide outpatient care to patients with cancer. Employees who did not provide outpatient care and trainees were not eligible. This study employed an implied consent process. Completion of the surveys was taken as the participants’ consent to participate in the study. The invitation and reminder emails included information on the study’s purpose, risks and benefits of participation, estimated amount of time required to complete the survey, a link to voluntarily complete the survey online through the use of the software program, Qualtrics, and outlined their ability to withdraw from the study at any time. Surveys were completed anonymously.

Descriptive analyses of the survey results were conducted. Counts and proportions were provided for demographic or practice-related variables. Mean (SD) and median (range) were calculated for continuous variables. The differences in survey responses among the three profession groups were compared using Chi-squared tests or Fisher’s exact tests. Correlations between average practice and average confidence were calculated. In addition, univariate linear regression models were performed to examine the associations between the outcome variable ‘average practice score’ and factors of interest, such as ‘average confidence score’, profession, gender, and years in practice. Average practice score was the numeric average of all opioid safety practice question responses (never: 1, rarely: 2, often: 3, always: 4). Average confidence score was the numeric average of all confidence question responses (assigned as strongly disagree: -2; somewhat disagree: -1; neutral: 0; somewhat agree: 1; strongly agree: 2). Variables showing statistical importance in the univariate analyses (*p* < 0.10), or with clinical importance, were entered into the multiple linear regression model. Statistical significance defined as *p*-value 0.05. SAS 9.4 (SAS Institute, Cary, NC, USA) was used to perform the statistical analyses.

## 3. Results

Given the survey length, we will present findings focused on demographics, attitudes towards opioid use in cancer care, confidence and practices in managing opioids in cancer care. The surveys were distributed to approximately 400 clinicians in January 2019, based on best estimates of staffing numbers within the cancer center. Sixty-five responses were received, including incomplete surveys; a response rate of 16%. Of these, 27 were MD/NP (41%), 31 were RN/CNS (48%), and 7 were pharmacists (11%). Thirty-five (54%) participants had been working in oncology for 10 years or less (Table 1).

Over a third of respondents (37%) agreed that patients with cancer are at very low risk for opioid-related harms because they have pain. Fifty-three percent agreed that patients with cancer frequently do not take their opioids as prescribed. Fifteen percent agreed that patients with cancer frequently become addicted to opioids. Twenty-seven percent agreed that many patients with cancer have comorbidities that put them at high risk for developing OUD. Twenty-five percent agreed that many patients with cancer are at high risk of opioid overdose.

**Figure 1:**
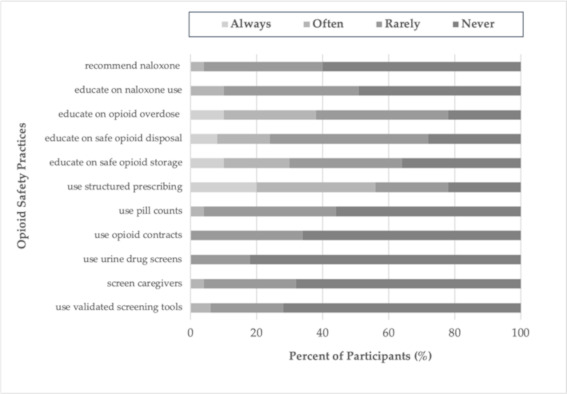
Frequency of opioid safety practices by percentage of participants (*n* = 51).

While a majority of respondents felt confident assessing patients’ pain while on opioids (75%), or providing pain management for a patient on opioid agonist therapy (OAT) (74%), only 55% felt confident managing aberrant medication taking behaviors, 39% in recognizing signs of OUD and only 47% in screening for OUD (Table 2). In terms of opioid safety practices, over half of participants never recommended or educated patients around naloxone use, utilized screening tools, pill counting, or urine drug screens. Over 60% rarely or never provided education on the safe storage or disposal of opioids (Figure 1).

Univariate linear regression analyses results demonstrated that practice score was significantly associated with confidence score and profession, whereas gender and years in palliative and oncology practice did not show significant effects. In the multiple linear regression model, higher confidence remained significantly associated with higher practice score (*β* = 0.38, *p* < 0.0001). Pharmacists reported higher overall practice scores compared to RN/CNS (*β* = 0.52, 95% CI = (0.16–0.88), *p* = 0.0052) and a trend toward higher scores compared to MD/NP (*β* = 0.35, 95% CI = (−0.01–0.71), *p* = 0.06).

## 4. Discussion

Increasingly, evidence suggests that patients with cancer might be at higher risk of NMOU and opioid-related harm than was previously thought [5,6,7,13]. However, there is limited understanding of how well-equipped oncology professionals are in recognizing, mitigating and managing these behaviors and associated risks.

Our results show that over one third of participants agreed that patients with cancer are at very low risk for opioid-related harms. This is raises concern that the belief that opioid-related harms are low in cancer population remains persistent, despite a paucity of supporting research [3,4]. Almost half of participants endorsed a lack of confidence in recognizing OUD in their patients with cancer. These results suggest that this under-recognition of OUD may be due to knowledge and practice gaps among oncology clinicians [5].

Participants in this study endorsed overall limited confidence in opioid management and risk mitigation practices, as well as limited and varied opioid safety practices. Participants infrequently engaged in safety practices such as screening, structured prescribing, pill counts, urine drug screens, overdose education, and naloxone education. This is not surprising given there are limited evidence-based recommendations for safer opioid prescribing in patients with cancer related pain [13]. 

Over 25% of participants reported never educating patients on safe opioid storage and disposal (Figure 1). A study by Reddy et al. found that 74% of adult cancer outpatients receiving opioids were unaware of proper opioid disposal methods, and that 46% have unused opioids at home [16]. Based on our results, we suspect that a lack of clinician education regarding safe storage and disposal is likely contributing to these unsafe conditions, which may lead to diversion, accidental overdose and death. Pharmacists demonstrated overall higher average practice scores, which may be due to the standardization of their medication safety education and practices and an integration with patient care at the time of opioid dispensing.

There are multiple limitations of this study. The sample size was small and from a single, tertiary cancer center. Dissemination of the survey via email, and the lack of an incentive to complete the survey, may have contributed to the poor response rate. Participants were mostly MDs and RNs with a smaller number of pharmacists. As such, our results may not be generalizable to a nonspecialist setting.

## 5. Conclusions

This study suggests that attitudes, confidence and practices around opioid safety in patients with cancer seem to widely vary between oncology care professionals. Overall, confidence in managing and mitigating risk of opioids appears to be low and safety practices, infrequent. This raises concern that there may be gaps in knowledge, education and training of oncology clinicians in the area of opioid safety for this unique patient population. This study provides us insight into these gaps that may be contributing to unsafe opioid prescribing, uncontrolled cancer pain, under-recognition of NMOU and OUD, and unnecessary risk. There is an urgent need for research, guidelines, and educational tools aimed at effectively and sustainably closing these gaps in order to minimize opioid-related harm while optimally managing symptoms and quality of life in patients with cancer. Larger, multicenter studies are needed to confirm our findings and inform future work in this area.

## Figures and Tables

**Table 1:** Demographic characteristics of survey participants (*n* = 65).

Profession	Number of Participants (%)
Physician	23 (35%)
Nurse Practitioner	4 (6%)
Registered Nurse/Clinical Nurse Specialist	31 (48%)
Pharmacist	7 (12%)
**Gender**	
Male	16 (25%)
Female	49 (75%)
Other	0
**Years working in oncology or palliative care**	
0–5	24 (37%)
6–10	11 (17%)
11–15	8 (12%)
16 or more	22 (34%)

**Table 2:** Level of confidence in opioid management and risk mitigation by number and percentage of participants (*n* = 51).

Area of Confidence (I am confident in…)	Agree *n* (%)	Neutral *n* (%)	Disagree *n* (%)
Assessing patients on opioids for cancer pain management	38 (75)	9 (18)	4 (8)
Providing pain management for a patient with OUD	34 (69)	7 (14)	9 (18)
Providing pain management for a patient on OAT*	38 (74)	7 (14)	6 (12)
Identifying AMTB*	11 (22)	15 (29)	25 (49)
Reviewing/upholding/negotiating an opioid contract	31 (61)	8 (16)	12 (23)
Recognizing signs of an OUD*	20 (39)	6 (12)	25 (49)
Screening for risk of OUD*	24 (47)	11 (22)	16 (31)
Managing AMTB*	28 (55)	14 (27)	9 (18)
Interpreting/collecting urine drug screens	26 (51)	6 (12)	19 (37)
Counselling around safe storage and disposal of opioids	17 (33)	5 (10)	29 (57)
Identifying patients with cancer at high risk of opioid overdose	21 (41)	8 (16)	22 (43)
Educating patients/caregivers on naloxone use	26 (51)	13 (25)	12 (24)

* OUD = opioid use disorder; OAT = opioid agonist therapy (e.g., buprenorphine/naloxone); AMTB = aberrant medication taking behaviors.

## Supplementary Materials

The following are available online at https://www.mdpi.com/1718-7729/28/1/86/s1, Oncology Opioid Safety Survey Template for Pharmacists.
