# Supplementary material for: Managing Opioids and Mitigating Risk: A Survey of Attitudes, Confidence and Practices of Oncology Health Care Professionals
Source: Curr Oncol. 2021 Feb 12;28(1):873–8. doi: 10.3390/curroncol28010086 (PMC7985764; doi:10.3390/curroncol28010086)
Supplement: Supplementary file 1 [file curroncol-28-00086-s001.pdf]

Supplemental Material: Oncology Opioid Safety Survey Template for Pharmacists

Managing opioids and mitigating risk:
A survey of attitudes, confidence and practices of oncology health care professionals

Pharmacist Survey

Section A: Q1-8 Demographics

1. Clinical practice area: Outpatient / Ambulatory Clinic (Please specify which clinical area: ______________)

2. Gender: M / F / Other / Prefer not to say

3. Years working as a pharmacist: 0-5 / 6-10 / 11-15 / 16 or more

4. Years working in oncology or palliative care: 0-5 / 6-10 / 11-15 / 16 or more

5. What percentage of outpatients in your practice are receiving opioids?
<5% / 5-10% / 11-25% / 26-50% / 51-75% / >75%

6. What percentage of outpatients in your practice use Illicit[A] opioids?
<5% / 5-10% / 11-25% / 26-50% / 51-75% / >75%

7. What percentage of outpatients in your practice have a known opioid use disorder[B]?
<5% / 5-10% / 11-25% / 26-50% / 51-75% / >75%

8. What percentage of outpatients in your practice are engaging in aberrant medication taking behaviors[C] of opioids?
<5% / 5-10% / 11-25% / 26-50% / 51-75% / >75%

A: *Illicit opioids are those which are classified as illegal (i.e.: heroin) or are purchased "on the streets" (i.e.: fentanyl, percocets) or are obtained from others.*
B: *Opioid use disorder is a medical condition defined by the Diagnostic and Statistical Manual of Mental Disorders, 5th edition (DSM-5) as "a problematic pattern of opioid use leading to clinically significant impairment or distress" that is manifested by specific clinical features (i.e. recurrent opioid use in situations in which it is physically hazardous).*
C: *Aberrant medication taking behaviors refer to any use of a prescription opioids in a manner other than as intended by the prescribing physician and pharmaceutical manufacturer. These behaviors can include unprescribed dose escalation, route alteration, procuring from other sources and diversion.*

Section B: Q 10-18 General Attitudes Towards Opioid Use in Cancer Care

Please indicate the extent of your agreement with each of the statements below:

	Strongly Agree	Somewhat Agree	Neutral	Somewhat Disagree	Strongly Disagree
Patients with cancer are at very low risk for harm related to opioids because they have pain					

Patients with cancer frequently become addicted to opioids					
Patients with cancer frequently take opioids as a means of "chemical coping"[A]					
Patients with cancer frequently do not take their opioids as prescribed by their physician or nurse practitioner					
Opioids should only be prescribed by pain specialists or palliative care physicians/nurse practitioners					
Many patients with cancer have comorbidities that put them at high risk for developing opioid adverse effects[B]<br><br>(If answered strongly agree or somewhat agree, follow-up question: What comorbidities do you think put patients with cancer at high risk for developing opioid adverse effects[B]?)					
Many patients with cancer have comorbidities that put them at high risk for developing opioid induced neurotoxicity[C]<br><br>(If answered strongly agree or somewhat agree, follow-up question: What comorbidities do you think put patients with cancer at high risk for developing opioid induced neurotoxicity[C]?)					
Many patients with cancer have comorbidities that put them at high risk for developing opioid use disorder[D]<br><br>(If answered strongly agree or somewhat agree, follow-up question: What comorbidities do you think put patients with cancer at high risk for developing opioid use disorder[D]?)					
Many patients with cancer are at high risk of opioid overdose[E]					

***A**: Chemical coping is the use of opioids to cope with emotional distress and is characterized by inappropriate and/or excessive opioid use*
***B:** Opioid adverse effects develop from the generalized non-specific action of opioids (i.e. constipation)*
***C:** Opioid induced neurotoxicity develop from opioid metabolites (i.e. myoclonus)*
***D:** Opioid use disorder is a medical condition defined by the Diagnostic and Statistical Manual of Mental Disorders, 5th edition (DSM-5) as "a problematic pattern of opioid use leading to clinically significant impairment or distress" that is manifested by specific clinical features (i.e. recurrent opioid use in situations in which it is physically hazardous).*

E: Opioid overdose occurs when opioids in high doses cause respiratory depression and death.

Section C: Q 19-35 Confidence in Managing Opioids in Cancer Care

Please indicate the extent of your agreement with each of the statements below:

	Strongly Agree	Somewhat Agree	Neutral	Somewhat Disagree	Strongly Disagree
I am confident in my clinical skills in assessing patients on opioids for cancer pain management					
I am confident in my clinical skills educating my patients about multi-modal pain management approaches					
I am confident in practicing the universal precautions[A] approach regarding opioid use					
I am confident in my clinical skills providing cancer pain management for a patient with a known opioid use disorder[B]					
I am confident in my clinical skills providing cancer pain management for a patient on opioid agonist therapy[C]					
I am confident in my clinical skills identifying aberrant medication taking behaviors[D] in my patients with cancer					
I am confident in my ability to manage aberrant medication taking behaviors[D] in my patients with cancer<br><br>(Follow-up question: How do you manage aberrant medication taking behaviours[D]?)					
I am confident in my ability to screen patients for risk of developing an opioid use disorder[B]					
I am confident in my ability to identify and appropriately refer patients with signs and symptoms of an opioid use disorder[B]					
I am confident in my skills interpreting urine drug screens					
I am confident in my ability to interpret and uphold an opioid contract[E]					
I am confident in counselling around safe storage and disposal of opioids					
I am confident in my clinical skills assessing for opioid adverse effects[F]					

I am confident in my clinical skills assessing for opioid induced neurotoxicity[G]					
I am confident in my clinical skills to identify patients with cancer at high risk of opioid overdose[H]					
I am confident in my clinical skills assessing for signs and symptoms of opioid overdose[H]					
I am confident in my clinical skills educating patients and/or family members and caregivers on naloxone use for a suspected opioid overdose[H]					

A: Universal precautions are ten steps used as a guide to manage chronic pain [1].
B: Opioid use disorder is a medical condition defined by the Diagnostic and Statistical Manual of Mental Disorders, 5th edition (DSM-5) as "a problematic pattern of opioid use leading to clinically significant impairment or distress" that is manifested by specific clinical features (i.e. recurrent opioid use in situations in which it is physically hazardous).
C: Opioid agonist therapy includes buprenorphine-naloxone (suboxone) and methadone maintenance therapy.
D: Aberrant medication taking behaviors refer to any use of a prescription opioids in a manner other than as intended by the prescribing physician and pharmaceutical manufacturer. These behaviors can include dose escalation, route alteration, procuring from other sources and diversion.
E: Opioid contracts are written agreements between the physician or NP and patient that outline the conditions of opioid prescribing.
F: Opioid adverse effects develop from the generalized non-specific action of opioids (i.e. constipation)
G: Opioid induced neurotoxicity develops from opioid metabolites (i.e. myoclonus)
H: Opioid overdose occurs when opioids in high doses cause respiratory depression and death.

Section D: Q 36-57 Practices in Managing Opioids in Cancer Care

Please indicate how often you practice the following statements:

	Always	Often	Rarely	Never
I conduct a thorough review of my patients' substance use histories				
I call my patients' pharmacies to gather more information about their opioid use history				
I call my patients' other providers (i.e.: family physician) for collateral information about their opioid use history				
I use Connecting Ontario to gather more information about my patients' opioid use history				
I assess where my patients are storing their opioids				
I assess how my patients are disposing their unused opioids				

I use validated screening tools[A] to screen patients with cancer I suspect are at high risk for opioid use disorder[B]<br><br>(If answered always, often or rarely, follow-up question: Please specify which of the following screening tools you use:<br>___ CAGE<br>___ ORT (Opioid Risk Tool)<br>___ SOAP-SF (Screener and Opioid Assessment for Patients with Pain– short form)<br>___ SOAPP-revised (SOAPP-R)<br>___ Other: Please specify ____________)				
I screen informal caregivers (i.e. family and friends) for risk of an opioid use disorder				
I review urine drug screens<br><br>(If answered always, often or rarely, follow-up question: For which patients do you review urine drug screens?)				
I participate in the care of patients with opioid contracts[C]				
I conduct pill counts				
I recommend the use of structured prescribing of opioids (i.e.: frequent dispensing and limited quantity with each prescription)				
I provide education to my patients regarding the safe storage of opioids				
I provide education to my patients regarding safe disposal of opioids				
I provide education to my patients regarding the safe use of breakthrough analgesia[D]				
I provide education to my patients on the management of opioid adverse effects[E]				
I provide education to my patients on the signs and symptoms of opioid induced neurotoxicity[F]				
I provide education to my patients about potential pharmacodynamic drug interactions when using opioids concomitantly with other CNS depressants (i.e.: benzodiazepines, alcohol, etc)				
I provide education to my patients about the signs and symptoms of opioid overdose[G]				
I provide education to my patients about naloxone use				
I recommend/dispense naloxone to patients at high risk of opioid overdose[G]				

I advocate for the involvement of addictions medicine in the care of my patients with cancer				

A: Examples of validated screening tools include CAGE, ORT (Opioid Risk Tool), SOAP-SF (Screener and Opioid Assessment for Patients with Pain– short form), SOAPP-revised (SOAPP-R). Note that these tools have been validated in non-cancer pain.
B: Opioid use disorder is a medical condition defined by the Diagnostic and Statistical Manual of Mental Disorders, 5th edition (DSM-5) as "a problematic pattern of opioid use leading to clinically significant impairment or distress" that is manifested by specific clinical features (i.e. recurrent opioid use in situations in which it is physically hazardous).
C*: Opioid contracts are written agreements between the physician or NP and patient that outline the conditions of opioid prescribing.*
D: Breakthrough analgesia refer to opioid doses used for management of transitory exacerbations of pain that occurs on the background of otherwise stable pain on opioid therapy.
E: Opioid adverse effects develop from the generalized non-specific action of opioids (i.e. constipation)
F: Opioid induced neurotoxicity develops from opioid metabolites (i.e. myoclonus)
G: Opioid overdose occurs when opioids in high doses cause respiratory depression and death.

Section E: Q 58-60 Personal Experience with Adverse Events in Cancer Patients Using Opioids

58. Over the past year, how many of your patients have had a significant adverse event (i.e.: delirium, respiratory depression, death) related to prescribed opioids?

None 1 2 3 4 5 or more

If none is selected, questions 59 to 60 will not appear.

59. Please provide examples of adverse events experienced by your patients or their caregivers:

60. Which of the following factors may have contributed to these adverse events (select all that apply)

___ The prescribed dose was too high
___ The patient accidentally took an incorrect dose
___ The patient deliberately took an incorrect dose
___ Dispensing error
___ Patient took the medication in a non-prescribed route (i.e.: crushed, injected, snorted)
___ Dose was not adjusted for co-morbidities (i.e.: renal or liver dysfunction)
___ Patient used opioids that weren't prescribed by their physician or nurse practitioner
___ Patient was using other illicit substances
___ Patient was using other CNS depressants (i.e.: benzodiazepines, alcohol, etc)
___ Lack of communication between patient and health care team
___ Other (Please specify:____________________________)

Section F: Q 61-72 Education and Resources

Please indicate the extent of your agreement with each of the statements below:

	Strongly Agree	Somewhat Agree	Neutral	Somewhat Disagree	Strongly Disagree
I am aware of community resources available for my patients if they exhibit aberrant medication taking behavior[A]					
I am aware of UHN resources available for my patients if they exhibit aberrant medication taking behavior[A]					
I am aware of community resources available for my patients if they have an opioid use disorder[B]					
I am aware of UHN resources available for my patients if they have an opioid use disorder[B]					
I am aware of community resources available for my patients if they are at high risk of opioid overdose[C]					
I am aware of UHN resources available for my patients if they are at high risk of opioid overdose[C]					

A: Aberrant medication taking behaviors refer to any use of a prescription opioids in a manner other than as intended by the prescribing physician and pharmaceutical manufacturer. These behaviors can include unprescribed dose escalation, route alteration, procuring from other sources and diversion.
B: Opioid use disorder is a medical condition defined by the Diagnostic and Statistical Manual of Mental Disorders, 5th edition (DSM-5) as "a problematic pattern of opioid use leading to clinically significant impairment or distress" that is manifested by specific clinical features (i.e. recurrent opioid use in situations in which it is physically hazardous).
C: Opioid overdose occurs when opioids in high doses cause respiratory depression and death.

67. Over the past year, have you participated in any educational activities or events on safe use of opioids for cancer pain management? Yes / No
If no is selected, question 68 will not appear.
68. If yes, in which of the following educational activities did you participate? (select all that apply):

Hospital rounds	
Conference	
Workshop or webinar	
Literature reading	
Pharma-sponsored events	
Other (please specify)	

69. Over the past year, have you participated in any educational activities or events on opioid use disorder in the context of patients with cancer pain? Yes / No
If no is selected, question 70 will not appear.

70. If yes, in which of the following educational activities did you participate? (select all that apply):

Hospital rounds	
Conference	
Workshop or webinar	
Literature reading	
Pharma-sponsored events	
Other (please specify: ________________)	

71. Rank each of the following from 1 to 11 (1 = most helpful, 11 = least helpful) in terms of how helpful they might be in your management of patients with cancer pain:

In-hospital workshop on opioid prescribing	
Online webinar on opioid prescribing	
Educational module on opioid prescribing	
Pocket guide on opioid prescribing	
Online institutional guidelines for opioid prescribing	
Help-line with telephone advice from pain specialist	
Help-line with telephone advice from palliative care physician	
Help-line with telephone advice from addictions medicine physician	
Help-line with telephone advice from pharmacist	
Help line with telephone advice from an advanced practice nurse	
Other (please specify: _____________)	

72. Do you have any additional comments about opioid prescribing at the Princess Margaret Cancer Centre?

References

1. Gourlay, D.L.; Heit, H.A.; Alnahrezi, A. Universal Precautions in Pain Medicine: A Rational Approach to the Treatment of Chronic Pain. *Pain Med.* **2005**, *6*, 107–112.
